# Use of MALDI-TOF MS to Discriminate between Biofilm-Producer and Non-Producer Strains of *Staphylococcus epidermidis*

**DOI:** 10.3390/ijerph15081695

**Published:** 2018-08-09

**Authors:** Pina Caputo, Maria Chiara Di Martino, Brunella Perfetto, Francesco Iovino, Giovanna Donnarumma

**Affiliations:** 1 Department of Experimental Medicine, Section of Microbiology and Clinical Microbiology University of Campania “Luigi Vanvitelli”, 80138 Naples, Italy; pinacaputo@hotmail.it (P.C.); mariachiaradimartino@gmail.com (M.C.D.M.); brunella.perfetto@unicampania.it (B.P.); 2 Division of General Surgery, Department of Cardiothoracic and Respiratory Science, University of Campania “Luigi Vanvitelli”, 80138 Naples, Italy; francesco.iovino@unicampania.it

**Keywords:** Matrix-assisted, laser desorption ionization time-of-flight (MALDI-TOF) mass spectrometry (MS), biofilm, *Staphylococcus epidermidis*

## Abstract

For the management of Staphylococci coagulase-negative infection, often related to biofilm formation, rapid and accurate identification is necessary in choosing a correct antibiotic therapy. Matrix-assisted laser desorption ionization time-of-flight (MALDI-TOF) mass spectrometry (MS) is becoming increasingly important for bacterial identification over traditional methods. Our aim was to validate the use of MALDI to discriminate *Staphylococcus epidermidis* biofilm-producing strains. Clinical strains coming from suture wires were identified and their protein profiles were compared to that obtained from two ATCC reference strains (biofilm producer and non-producer). MALDI identified the eighteen isolates as *S. epidermidis*, combining sixteen profiles with the biofilm producer and two with the non-producer, confirming the results of crystal violet assay. Our data highlight that MALDI can be considered a good tool to discriminate between biofilm-producer and non-producer strains of *S. epidermidis*, thus helping to establish an effective antibiotic therapy.

## 1. Introduction

Clinical microbiology laboratories often use conventional methods, based on culture with selective media, analysis of microbial phenotypic characteristics, and biochemical tests for microbial identification. A rapid and accurate identification of microorganisms is necessary for the management of infectious diseases, particularly to choose an effective therapy. For this reason, the routine identification of bacteria and fungi by matrix-assisted laser desorption ionization time-of-flight mass spectrometry (MALDI-TOF MS) has become a true revolution in clinical microbiology laboratories [1]. This approach generates a mass spectrum with a number of defined protein peaks in the range of 70–200, for about 10^5^–10^6^ fresh cell biomass. The protein profiles obtained from the isolated microorganisms are compared against a database of profiles of well characterized reference isolates comprising 10 reference spectra per bacterial species and a 1.9 identification score (the Brucker system).

Biofilm is a key virulence factor for a wide range of microorganisms responsible for chronic infections. It can be described as a hardly penetrable, vast colony of microorganisms embedded in a highly hydrated exopolymer matrix. Following the establishment of a biofilm, its adhesive strength and viscoelastic properties make it very difficult to remove from surfaces. In addition the resident microorganisms become resistant to drugs and to host immune responses [2].

Staphylococci, mainly *Staphylococcus aureus* and *Staphylococcus epidermidis*, are well-known causative agents of a large number of human infectious diseases, including skin, soft tissue, respiratory tract, bone, joint, and endovascular infections, as well as infections related to implanted medical devices [3,4]. 

*S. epidermidis* is the most important member of coagulase-negative staphylococci and one of the most abundant colonizers of human skin [5]. For a long time it was considered as being innocuous, however, now it has been identified as an important opportunistic pathogen that can cause significant problems when breaching the epithelial barrier and in infections associated with indwelling medical devices, especially because of its ability to form a biofilm [3,6]. The main components of a biofilm are the Polysaccharide Intercellular Adhesin (PIA) or the Polymeric *N*-acetyl-glucosamine (PNAG) produced by ica operon-encoded enzymes that include four specific genes (A, B, C, and D), a regulatory gene (*icaR*), and a transposable element, *IS256* [7,8]. PIA plays an essential role in the initial bacterial adherence to surfaces and in intercellular adhesion during the cells aggregate formation [9,10]. Despite the role of the *ica locus* in staphylococcal biofilm development, scientific evidence has demonstrated the existence of PIA/PNAG-independent biofilm mechanisms [9]. Different protein factors in ica-independent biofilm mechanisms have been identified, including specific surface proteins such as the accumulation-associated protein Aap and Bhp. The latter is the Bap homologue, which is a biofilm-associated protein in *S. aureus* [11,12,13].

Although infections caused by biofilm-producer *S. epidermidis* strains are increasing, no commercially innovative options for the identification of these strains are available. Rapid identification of these is essential to develop an effective and correct therapeutic plan for the resolution of infection.

MALDI-TOF MS has emerged as a rapid, inexpensive, and accurate method for bacterial identification [14]. It may be an acceptable alternative to conventional identification methods and for some species it can be more efficient. In this study, we analyzed the protein profiles obtained by MALDI-TOF MS of eighteen strains of *S. epidermidis* coming from suture wires, already identified in a previous work with conventional methods [15]. The spectra obtained were compared with a personalized database; this included two main profiles obtained from the analysis of 60 spectra of two ATCC reference strains, one *S. epidermidis* strain known as a biofilm producer and one *S. epidermidis* strain known as a non-producer. The analysis has highlighted that MALDI-TOF MS is an effective option for the fast and direct discrimination of biofilm-forming strains, this would be enhanced through introducing known protein profiles of biofilm-producer and non-producer strains into the database. The direct identification, after isolation, of a bacteria strain as a biofilm producer would allow the development of a valid and effective therapeutic plan.

## 2. Materials and Methods

### 2.1. Bacterial Strains 

A collection of 18 strains of *S. epidermidis* isolated from 40 colonies from 119 suture wires from the Division of General Surgery, Department of Cardiothoracic and Respiratory Science, University of Campania “Luigi Vanvitelli” were used for this study. The bacterial strains, identified in a previous work with ViteK2 (bioMerieux) [15], were numbered from SE01 to SE18. Two control strains from ATCC were also used, one ATCC 12,228 non-biofilm producer and the other ATCC 35,984 biofilm producer. The ATCC 35,984 biofilm-producer strain presents in its genome some genes that are specifically activated for biofilm production. These include the *Ica* gene complex, responsible for the production of enzymes involved in the synthesis of PIA; genes involved in the production of Microbial Surface Components Recognizing Adhesive Matrix Molecules (MSCRAMM), adhesin proteins able to interact with host matrix proteins that cover abiotic surfaces such as medical devices; and the *bhp* gene, responsible for the production of Bhp (Bap homolog protein) also involved in biofilm adhesion. These genes are absent in the genome of *S. epidermidis* ATCC 12,228, a non-biofilm producer.

### 2.2. Biofilm Assessment 

Samples and controls were tested for their ability to form biofilm. Overnight cultures of strains in Tryptic Soy Broth (TSB) were diluted to a concentration of 10^7^ CFUs/mL, and aliquots (200 μL) of the diluted bacterial suspension were placed into 96-well flat-bottomed sterile polystyrene microplates (Corning-Costar) and incubated overnight at 37 °C in aerobic conditions. The biofilm formed was quantified by crystal violet assay, performed as described by Xu et al. (2016) and Di Domenico et al. (2016) with some modification [16,17]. After 24 h, the attached bacteria were washed twice with 200 μL of phosphate buffered saline (PBS) and air-dried for 45 min. The wells were then stained with 200 μL of 1% aqueous crystal violet solution for 45 min, and were rinsed with 200 μL of sterile distilled water to remove excess dye and air-dried. The dye associated with the attached biofilm was dissolved in a solution of 200 μL of ethanol, and the absorbance was measured at OD 570/655 nm on a micro plates reader 580 Bio-Rad (Bio-Rad, Hercules, CA, USA). 

### 2.3. Scanning Electron Microscopy of S. epidermidis Colonized Wires

Samples of threads gauge 2 (Coated, Braided Lactomer 9-1) colonized by *S. epidermidis*, were fixed in paraformaldehyde (4%) in phosphate saline buffer over night and then dehydrated with increasing ethanol percentage (30–90% in water for 5 min, twice 100% for 15 min). The samples were then treated in a Critical Point Dryer (EMITECH K850, Quorum technologies Ltd., Laughton, East Sussex, UK) sputter coated with platinum-palladium (Denton Vacuum DESKV, Denton Vacuum, Moorestown, NJ, USA) and observed with Supra 40 FE-SEM (Zeiss, Germany).

### 2.4. MALDI-TOF MS S. epidermidis Spectra Analysis 

Mass spectrometric experiments were performed with the spectrometer MALDI-TOF mass produced and marketed by Bruker Daltonics (Germany). A pulsed laser at a wavelength of 337 nm was used for the ionization. The analysis of the data obtained was performed with MALDI software Biotyper RTC (Bruker Daltonics, Bremen, Germany), FlexiControl, FlexiAnalysis (Bruker Daltonics, Bremen, Germany), MALDI Biotyper 3.0 (Bruker Daltonics, Bremen, Germany).

For MALDI-TOF analysis, all samples (clinical isolates and ATCC strains) were subjected to formic-acid extraction to improve the test quality [18]. Nine hundred microliters of ethanol absolute were added to 300 µL of sterile deionized water containing one isolate colony of the analyzed strain cultured in BHI agar, the suspension was centrifuged for 5 min at 13,000 rpm. The pellet was air-dried and was re-suspended in 70 µL of 70% formic acid solution. An equivalent amount of pure acetonitrile was added to suspension. After mixing, the samples were centrifuged for 5 min at 13,000 rpm, for protein extraction from the pellet. One microliter of supernatant was pipetted onto a MALDI plate and the samples were completely dried at room temperature. Before proceeding with the analysis, 1 μL of α-HACCA matrix was added.

The analysis was performed using the MicroFlex tool (Bruker Daltonics, Bremen, Germany), used in positive linear mode in the range of 2000–20,000 *m*/*z*. The obtained mass spectra were automatically acquired according to the system procedure, which identified the various microorganisms attributing to each of them a score related to degree of similarity with the reference spectrum contained in the database. For each clinical isolate, mass spectra were obtained with FlexControl Softwares (Bruker Daltonics, Bremen, Germany).

A personalized database was created with the two ATCC reference strains. For this reason, sixty mass spectra of the ATCC reference strains of *S. epidermidis* 12,228 (non-biofilm producer) and ATCC 35,984 (biofilm producer) were collected to obtain two Main Spectra Profiles (MSPs) or reference spectra.

MALDI Biotyper 3.0 software according to the protocol provided by Bruker Daltonics was used to associate the spectra of samples to one of the two MSPs created, based on the common characteristics in the mass spectra. The association of a sample to an MSP is based on the same algorithm used to identify microorganisms from the MALDI BioTyper RTC software; the association of the spectrum under examination and the reference spectrum occurs by comparing both *m*/*z* and intensity values obtained individually in the two spectra.

For each sample, the software generated a graphic representation as a result of the association between the “unknown” sample and the reference MSP.

## 3. Results

### 3.1. Biofilm Assessment with Crystal Violet Assay

**Figure 1:**
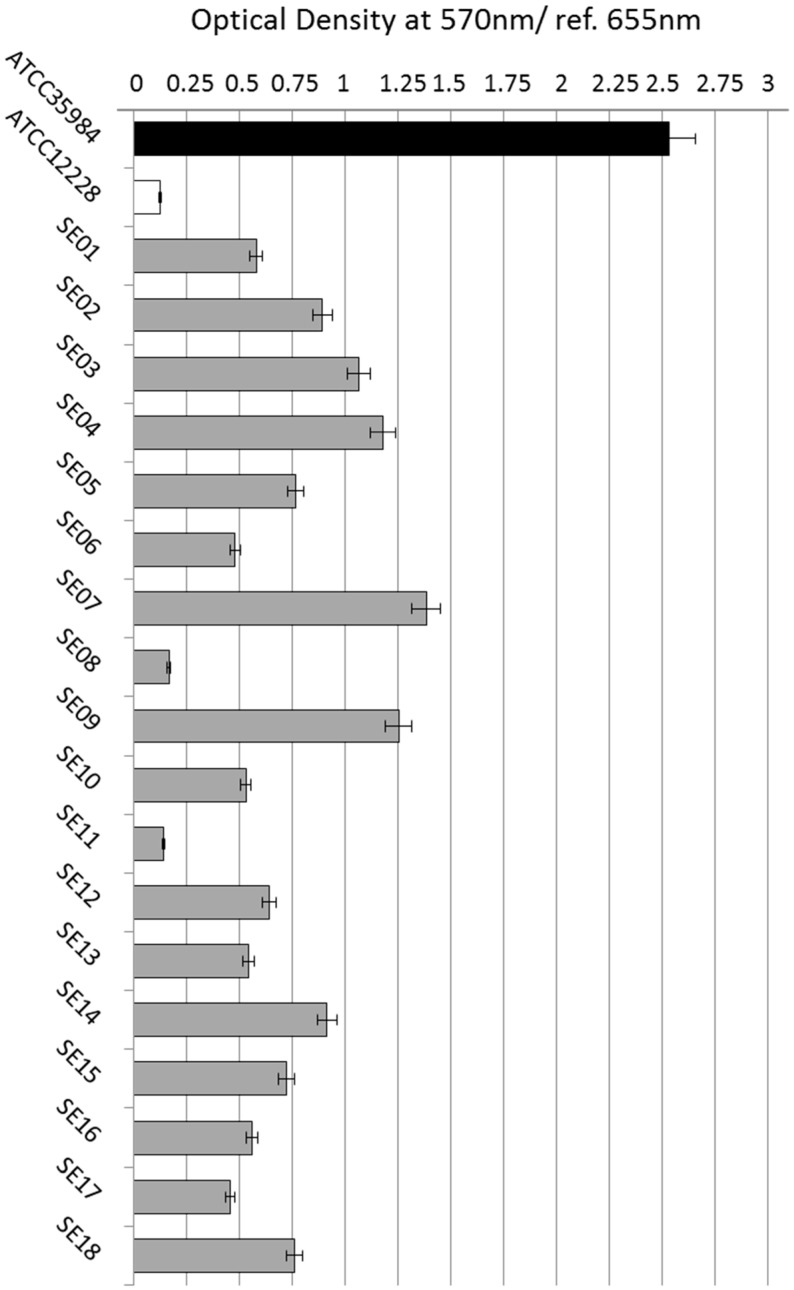
*Staphylococcus epidermidis* biofilm assessment. Four isolates showed a high-level of biofilm formation (biofilm index ≥ 1) (SE03, SE04, SE07, and SE09); ten moderate biofilm formations (biofilm index ≥ 0.5) (SE01, SE02, SE05, SE10, SE12, SE13, SE14, SE15, SE16, and SE18); two showed a low-level of biofilm formation (biofilm index < 0.5) (SE06 and SE17); and two showed a level overlapped to *S. epidermidis* ATCC 12,228 non-biofilm producer (SE08 and SE11).

As shown in Figure 1, the reference strain ATCC 35,984 produces a great amount of biofilm, unlike the strain ATCC 12,228. Sixteen of the eighteen strains tested proved to be biofilm producers, in fact they produced biofilms equal to or smaller than the strain ATCC 35,984. We established an arbitrary index to indicate the rate of biofilm production, in relation to the optical density obtained in crystal violet assay. Therefore, the clinical isolates SE03, SE04, SE07, and SE09 showed a high-level of biofilm formation (biofilm index ≥ 1); SE01, SE02, SE05, SE10, SE12, SE13, SE14, SE15, SE16, and SE18 showed a moderate biofilm formation (biofilm index ≥ 0.5); SE06 and SE17 showed a low-level of biofilm formation (biofilm index < 0.5); and SE08 and SE11 showed a level overlapped to *S. epidermidis* ATCC 12,228 non-biofilm producer.

### 3.2. SEM of Colonized Suture Wires

**Figure 2:**
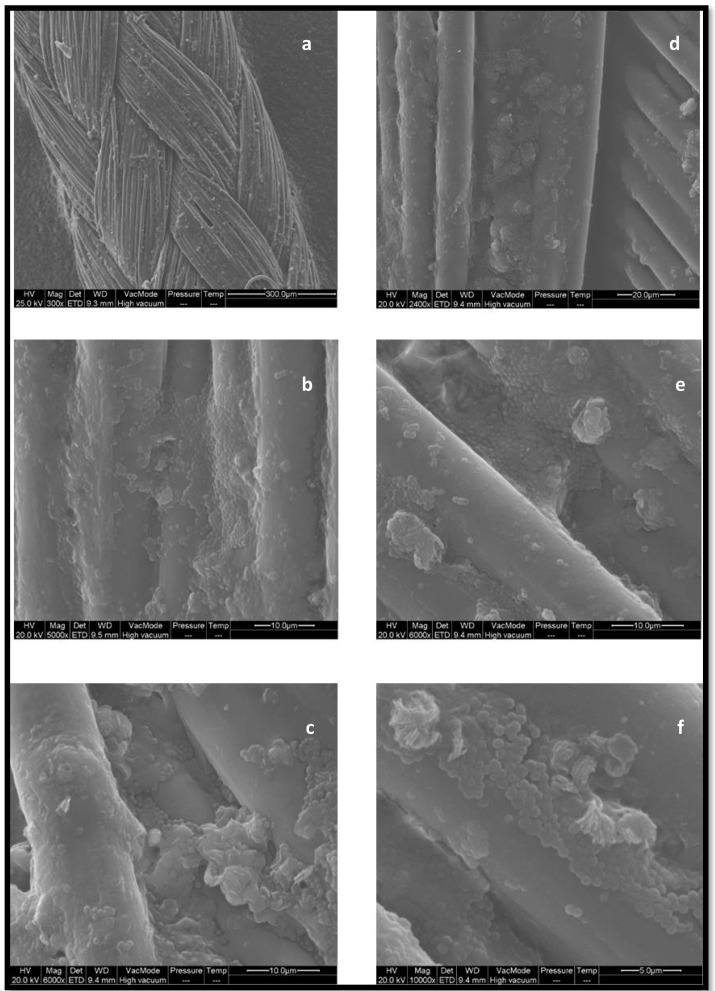
Gauge 2 suture thread (Coated, Braided Lactomer 9-1) colonized by *S. epidermidis* biofilm observed under SEM with increasing magnification (**a**,**b** Mag. 5000×; **c**–**e** Mag. 6000×; **f** Mag. 10,000×). The images evidence a well-developed microcolony, surrounded by regions of multilayered cells in the relatively flat biofilm.

Figure 2 shows a suggestive example of biofilm formation by *S. epidermidis* on a gauge 2 synthetic absorbable suture thread (Coated, Braided Lactomer 9-1), seen under SEM with increasing magnifications.

### 3.3. MSPs of S. epidermidis Reference Strains with MALDI-TOF MS 

**Figure 3:**
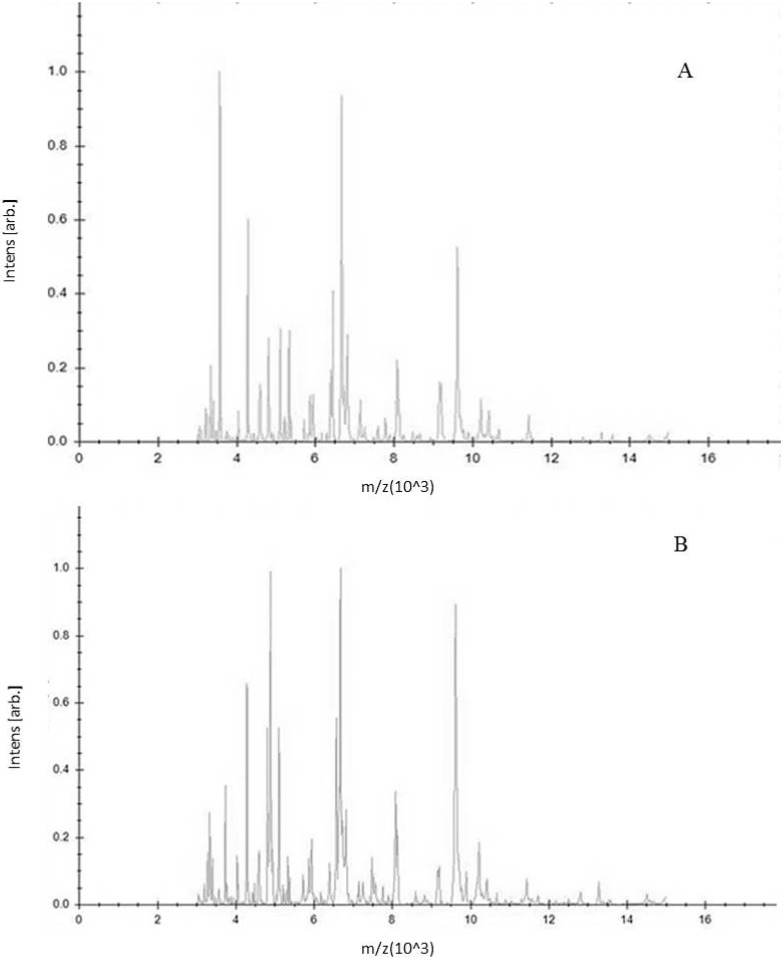
(**A**) Main Spectrum Profile of *S. epidermidis* ATCC 12,228 (non-biofilm producer); (**B**) main Spectrum Profile of *S. epidermidis* ATCC 35,984 (biofilm producer). They were obtained as a mean value of sixty mass spectra using MALDI Biotyper 3.0 software. The *m*/*z* ratio is shown on the axis of the abscissas, the intensity values is shown on the axis of the ordinates.

A Main Spectrum Profile is a reference spectrum consisting of a series of reference peaks corresponding to a single bacterial species. MSPs were created by analyzing 60 spectra obtained from samples subjected to the same growth conditions. Figure 3A,B show the two different mass spectra of strains of *S. epidermidis* ATCC 12,228 (non-biofilm producer) and ATCC 35,984 (biofilm producer), respectively. A customer database containing these two MSPs was created.

### 3.4. Samples Spectra Analysis

For each clinical isolate, MALDI-TOF MS confirmed the previous identification, all samples were identified as *S. epidermidis* with a score greater than 2, a highly reliable analysis index, and generated a spectrum.

The mass spectra obtained from the clinical strains were compared with the ATCC main spectra profiles using MALDI Biotyper 3.0 software, giving a graphic representation.

**Figure 4:**
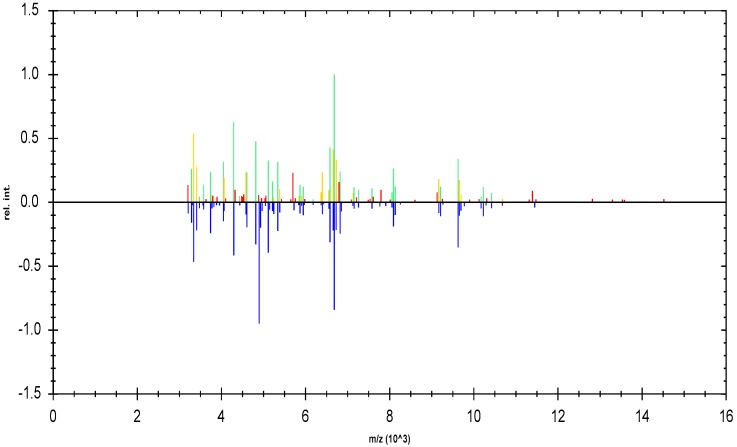
Graphic representation of identification of *S. epidermidis* (clinical strain) as *S. epidermidis* biofilm producer. In the upper half of the graph, the normalized peak list spectrum of the unknown sample is shown (*S. epidermidis* SE05). In the lower half of the graph, the peak list spectrum of the MSP *S. epidermidis* 35,984 selected by MALDI Biotyper 3.0 is shown.

**Figure 5:**
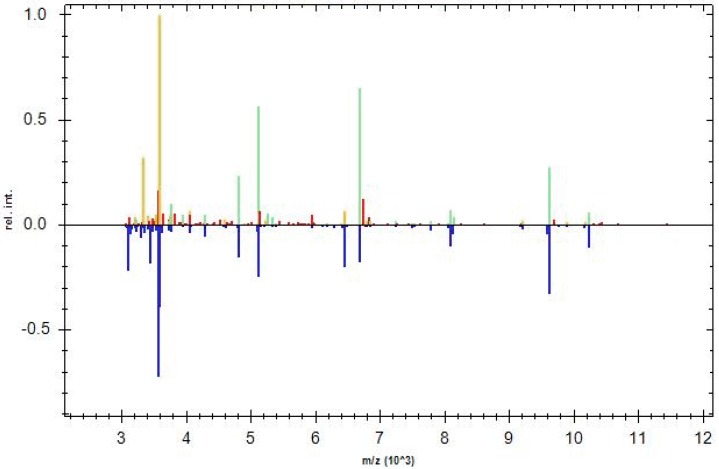
Graphic representation of identification *of S. epidermidis* (clinical strain) as *S. epidermidis* non-biofilm producer. In the upper half of the graph, the normalized peak list spectrum of the unknown sample is shown (*S. epidermidis* SE08). In the lower half of the graph, the peak list spectrum of the MSP related to *S. epidermidis* 12,228 selected by MALDI Biotyper 3.0 is shown.

Sixteen of the 18 clinical isolates were associated with the MPS of *S. epidermidis* ATCC 35,984 biofilm producer, among these sixteen strains, by crystal violet analysis, four proved to be high biofilm producers with a biofilm index of ≥1, ten moderate biofilm producers with a biofilm index of ≥0.5, and two low biofilm producers with a biofilm index of <0.5, but very close to 0.5. Among the strains that proved to be low biofilm producers two of them, which had shown OD values of less than 0.2, were associated with the MPS of *S. epidermidis* ATCC 12,228 non-biofilm producer. Figure 4 and Figure 5 show a representative example of a graphical comparison of a clinical isolate (SE05) associated with the MPS of the ATCC biofilm-producer strain, and of a clinical isolate (SE08) associated with the MPS of the ATCC non-producer strain.

The upper half of the graphic representation shows the normalized peaks list spectrum of the unknown sample, while the lower half shows the peaks list spectrum of the MSP to which the sample was associated by the software. The color of each peak of the sample reflects the closeness of the match to the reference MSP (green = full match, yellow = partial match, red = no match).

In the graphic representations there is a greater presence of green and yellow peaks than red peaks, indicating a greater correspondence of the clinical strains with MSPs of *S. epidermidis* reference strains.

## 4. Discussion

Matrix-assisted laser desorption ionization time-of-flight mass spectrometry becomes day by day a fast, accurate, and reliable tool for microbial identification. With this technology it is possible to obtain a protein profile, both from intact cells or cell extracts, and by comparing it to a database of bacterial reference mass spectra one can obtain a rapid identification of genus, species, and in some cases the sub species level [19]. In addition, recently, it has been increasingly used for the analysis of bacteria grown in biofilm [20,21]. Biofilm can be defined as a microbially-derived sessile community where cells are attached to each other and to a substratum embedded in a matrix of extracellular polymeric substance, and exhibit an altered phenotype regarding: growth, gene expression, and protein production [22]. Biofilms can cause serious medical problems since they represent a reservoir of bacteria that can be shed into the body, leading to chronic infections [23]. Biofilms play a pivotal role in healthcare-associated infections, especially those related to the implant of medical devices, such as catheters, orthopaedic implants, artificial heart valves, suture threads also represent a good carrier for biofilm formation [15,24,25]. Two main mechanisms contribute to biofilm resistance: (1) prevention of the antibacterial substance from reaching its target, e.g., by limited diffusion or repulsion, and (2) the specific physiology of a biofilm, which limits the efficacy of antibiotics, mainly of those that target active cell processes, and which may also include specific subpopulations of resistant cells [6,26]. *S. epidermidis* and *S. aureus* are counted among the major biofilm-producing bacteria.

Staphylococcal biofilm accumulation can have two different mechanisms: firstly, a ica-dependent mechanism, supported by the polysaccharide intercellular adhesion production; related to the high molecular weight of poly-*N*-acetyl-ß-(1–6)-glucosamine (PNAG) which is synthesized by ica ADBC operon products [27,28] or a ica-independent mechanism. The ica operon is present in most *S. aureus* isolates but its expression is related to the strain and to the growth conditions [29,30].

The aim of our study was to demonstrate that MALDI-TOF MS can be used to discriminate *S. epidermidis* clinical isolates as biofilm producer, quickly and accurately, together with identification. For this purpose, a strain collection coming from contaminated suture threads was used.

In our previous study we showed that in 119 patients undergoing surgery, 83% showed a bacterial colonization of the suture threads. From the colonized suture threads, several different microorganisms were isolated, main part Gram-positive cocci. Among these, 18 strains of *S. epidermidis* were identified with Vitek2 (Biomerieux) [15], confirming that the suture threads provide a good reservoir for biofilm-forming bacteria.

These clinical isolates together with the two reference strains were tested with the crystal violet test for their ability to produce biofilm; all the strains showed a more or less evident biofilm production, only two showed a level overlapping with that obtained from the non-biofilm producer *S. epidermidis* reference strain. Our analysis, carried out with the use of MALDI, under standard sample preparation conditions, demonstrated the potential to distinguish between biofilm-positive and biofilm-negative strains.

The *S. epidermidis* ATCC 35,984 strain (biofilm producer) and the ATCC 12,228 strain (biofilm non-producer) were used to create two Main Spectrum Profiles by analyzing 60 spectra obtained with MALDI-TOF MS using MALDI Biotyper 3.0 software. The MPSs were used as reference spectra, in a personalized database, comparing them with those obtained from the eighteen clinical isolates of *S. epidermidis*. The MALDI-TOF MS assay confirmed the previous identification, recognizing the isolates as *S. epidermidis* strains, associating sixteen spectra of *S. epidermidis* samples to the MPS of *S. epidermidis* ATCC 35,984, thus identifying them as biofilm-producing strains, and two to the MPS of *S. epidermidis* ATCC 12,228 non-biofilm producer. Most of the strains associated with the MPS of the ATCC strain biofilm producer showed a high or moderate biofilm production during the biofilm assessment, while only two were low biofilm producers, with an OD next to 0.5. The two low biofilm producer strains with an OD of less than 0.2 were associated with the MPS of the ATCC non-biofilm producer strain. Although these data confirmed those obtained with the crystal violet assay, the response times were greatly reduced and the accuracy increased.

## 5. Conclusions

Our data highlight that matrix-assisted laser desorption ionization time-of-flight mass spectrometry, using the same software as was used in the identification stage, is able to discriminate between biofilm-producer and non-producer strains of *S. epidermidis* using a personalized database which includes specific protein profiles of strains certainly known as biofilm producer and non-producer. The MALDI association to one or the other strains seems to be related to the ability of the strain to produce biofilm under examination. In fact, only two of the eighteen strains, identified by traditional methods as low biofilm producers and exhibiting an OD <0.2 at crystal violet assay, were identified with MALDI as non-biofilm producers. The fast recognition of clinical isolates, able to cause serious clinical problems through the formation of biofilms, is an important key to establishing a correct and efficient antibiotic therapy and thus reducing the possible development of device-related infections.
